# Shockproof Deformable Infrared Radiation Sensors Based on a Polymeric Rubber and Organic Semiconductor H_2_Pc-CNT Composite

**DOI:** 10.3390/polym15122691

**Published:** 2023-06-15

**Authors:** Muhammad Tariq Saeed Chani, Khasan S. Karimov, Tahseen Kamal, Noshin Fatima, Mohammed M. Rahman, Abdullah M. Asiri

**Affiliations:** 1 Center of Excellence for Advanced Materials Research, King Abdulaziz University, Jeddah 21589, Saudi Arabia; 2 Ghulam Ishaq Khan Institute of Engineering Sciences and Technology, Topi 23640, Pakistan; 3 Center for Innovative Development of Science and Technologies of Academy of Sciences, Rudaki Ave., 33, Dushanbe 734025, Tajikistan; 4 Faculty of Engineering, Technology and Built Environment, UCSI University, Kuala Lumpur 56000, Malaysia

**Keywords:** polymeric rubber, surface-type structure, sandwich-type structure, impedance, temperature, shockproof devices

## Abstract

Polymeric rubber and organic semiconductor H_2_Pc-CNT-composite-based surface- and sandwich-type shockproof deformable infrared radiation (IR) sensors were fabricated using a rubbing-in technique. CNT and CNT-H_2_Pc (30:70 wt.%) composite layers were deposited on a polymeric rubber substrate as electrodes and active layers, respectively. Under the effect of IR irradiation (0 to 3700 W/m^2^), the resistance and the impedance of the surface-type sensors decreased up to 1.49 and 1.36 times, respectively. In the same conditions, the resistance and the impedance of the sandwich-type sensors decreased up to 1.46 and 1.35 times, respectively. The temperature coefficients of resistance (*TCR*) of the surface- and sandwich-type sensors are 1.2 and 1.1, respectively. The novel ratio of the H_2_Pc-CNT composite ingredients and comparably high value of the *TCR* make the devices attractive for bolometric applications meant to measure the intensity of infrared radiation. Moreover, given their easy fabrication and low-cost materials, the fabricated devices have great potential for commercialization.

## 1. Introduction

The sensing of infrared irradiation is crucial not only for the detection of different objects but also for security and monitoring purposes. There are two main types of detectors: photonic and thermal. IR radiation’s thermal effects may be detected through numerous temperature-dependent phenomena. A lot of research work has been performed in this area. The layer-structured bolometer, based on organic semiconductor diodes, was patented by Vogt et al. [1] for the measurement of temperature. It was found that these devices are particularly sensitive in the range of infrared radiation. In another report, the properties of a low-band-gap organic photodiode-based IR sensor with an up-converting phosphor were discussed [2]. The results revealed the sensing principle of the polymer-based organic photodiode in near-IR. The emerging designs and guidelines for the characterization of polymer-based IR photodetectors are described in detail elsewhere [3]. Moreover, it was mentioned that the primary focus of the present photodetection technology is on inorganic semiconductors, which are deposited through epitaxial growth. Similarly, Muller et al. patented organic- or polymeric-materials-based IR sensor systems and devices for the detection of infrared radiation at wavelengths measuring 9 to 3 µm [4]. These devices are suitable for the detection of a mammal’s movements in the forest. These narrowband near-IR photodetectors were fabricated using a blend of fullerene (acceptor) and organic semiconductors (donor) [5]. The optical cavity architecture of these devices boosted the photocurrent for wavelengths inside the intermolecular charge transfer absorption band. These zinc phthalocyanine–fullerene-based devices exhibited narrowband photodetection at wavelengths below the optical gap of the donor and acceptor with a spectral width below 36 nm. Near-IR ultra-flexible organic photodetectors for photoplethysmogram conformal sensors were designed [6]. These sensors contain bulk heterojunction photovoltaic layers made of regio-regular polyindacenodithiophene-pyridyl[2,1,3]thiadiazole-cyclopentadithiophene. An organic-materials-based photodetector and diode have been described as well [7,8]. An organic IR-sensitive LED (light-emitting diode) for multi-spectral imaging was presented in work by Lai et al. [9], where they used expensively grown III-V semiconductors as sensors.

The thin films of metal-free phthalocyanine were characterized for UV-to-near-infrared absorption spectra and structural properties [10]. The indirect onset and fundamental energy gaps were found to be 1.41 and 2.47 eV, respectively, while annealing showed a minor effect on the optical properties. For bolometric applications, the films of metallic and semiconducting single-walled CNT composites were studied, and it was found that the bolometer’s performance significantly depends on the morphology of the composite [11]. Cracked composite films with a highly aligned array of the SWNTs, which were suspended in a silicon substrate, showed a high sensitivity because of high thermal isolation. However, the uncracked composite films showed a lower sensitivity because of low thermal isolation instead of having a high thermal coefficient of resistance (α). The value of α was up to −6.5%/K. A graphene-based rapid-response room-temperature nano-mechanical bolometer was also studied, wherein a graphene-based nanoelectromechanical system was used for light detection through resonance sensing [12]. In the resonance sensing approach, a suspended graphene resonator was heated and tensed by absorbed light, which caused a change in its resonant frequency. An IR sensor, containing a specifically patterned Au black absorption layer, was fabricated via the CMOS process [13]. This thermopile or sensor comprises alternate regions of n-type and p-type polysilicon that are connected to a Si_3_N_4_ layer in a series. Semiconducting-materials-based pyroelectrics and bolometers were studied to investigate the parameters of infrared thermal detectors [14]. An image sensor composed of organic semiconductors was fabricated for sensing light at more than one wavelength [15]. Lee et al. discussed the current developments in the application of organic-materials-based sensors in the field of health (for self-monitoring systems) [16]. The importance of developing wearable organic-materials-based sensors that have the ability to identify the human body’s signals (biophysical signals) was pointed out in [16]. The IR absorption behavior of carbon nanotubes synthesized through CVD (chemical vapor deposition) was studied, and it was reported that synthesized CNTs are semiconductive with a 100 meV bandgap [17]. These properties are considered attractive for mid-IR sensor applications. A pyroresistive, highly sensitive infrared bolometer consisted of bilayer organic films [18]. This bolometer was fabricated by depositing a sub-micrometer-sized pyroresistive thin-layer of crystals on a polymeric thin film and was recommended to be used for passive infrared sensing and direct contact thermometers. In contrast to expensive inorganic semiconductors, a low-cost, conductive, polymer-based bolometer for infrared detection systems was presented in ref. [19]. This bolometer was based on a poly(3,4-ethylene-dioxythiophene) freestanding layer, which showed low thermal conductivity, high IR absorption, and good thermistor action. Self-oriented, single-walled, CNT-based films were electrically and thermally characterized for bolometric applications [20]. These films showed a high temperature coefficient and high sensitivity.

A review of the properties of CNT-reinforced elastomeric nanocomposites was presented by Zhang et al. [21]. They compared the effects of different types of CNTs on the mechanical and electrical properties of nanocomposites. As a result of these investigations, it was established that the properties of elastomeric nanocomposites are strongly affected by the filler type and its nature. Recently, we designed and fabricated graphene- and CNT-based multifunctional sensors [22]. These sensors were investigated for the measurement of pressure, displacement, and temperature gradients. It was found that with a 36 °C rise in the temperature gradient, the resistance rises, on average, by 1.53 times. This rise in temperature causes an increase in thermoelectric voltage non-linearly, up to 0.6 mV. However, a linear increase of up to 0.45 μÅ in thermoelectric current was observed.

Recently, the temperature- and humidity-sensing properties of graphene–carbon nanotube–silicone adhesive [23] nanocomposites were investigated by our group. These sensors were fabricated using doctor blade technology, and their sensing mechanism was based on the variation in impedance and resistance with temperature or humidity change. These sensors were tested for a temperature interval of 37 °C to 87 °C. The average impedance change in the rising temperature, from 37 °C to 87 °C, was up to −19.8 Ω/°C. The value of the *TCR* (temperature coefficient of resistance) for these sensors was up to −0.46%/°C.

It Is essential to develop various complex deformable and elastic devices, especially on the basis of polymeric and carbon-containing materials, which are usually environmentally friendly. In a continuation of our efforts in designing, fabricating, and investigating the properties of various organic–inorganic-composite-material-based sensors, here, we present the results of investigations of a shockproof deformable organic semiconductor (H_2_Pc) and carbon nanotube (CNT)-based IR bolometers. These devices were fabricated on polymeric rubber substrates (styrene–butadiene rubber). These substrates, because of their flexible nature, make the fabricated devices deformable and shockproof [24]. The comparison in this study, with the data available in the literature, showed that the investigated composite (H_2_Pc-CNT) of the presented ratio of components was not previously used for the fabrication of sensors. To the best of our knowledge, this H_2_Pc-CNT–rubber composite was studied for the first time for the fabrication of IR sensors. The devices are desirable for bolometric applications meant to measure the intensity of IR radiation given the *TCR*’*s* fairly high value. Additionally, the easy fabrication and involvement of inexpensive materials mean these produced devices have an excellent chance of becoming commercialized.

## 2. Experimental

### 2.1. Materials

**Figure 1:**
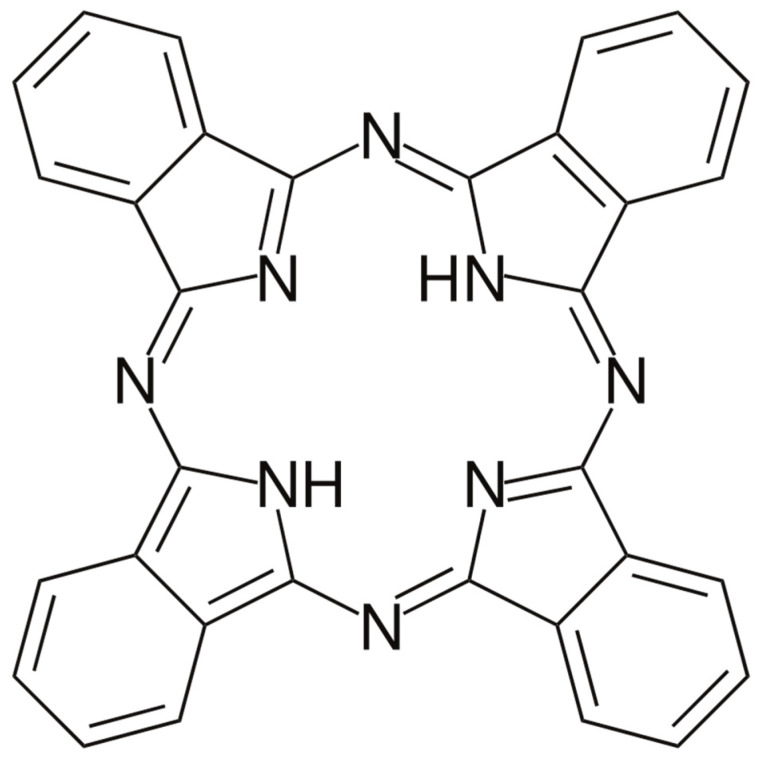
Molecular structure of organic semiconductor metal-free phthalocyanine (H_2_Pc) reprinted from Ref. [25].

**Figure 2:**
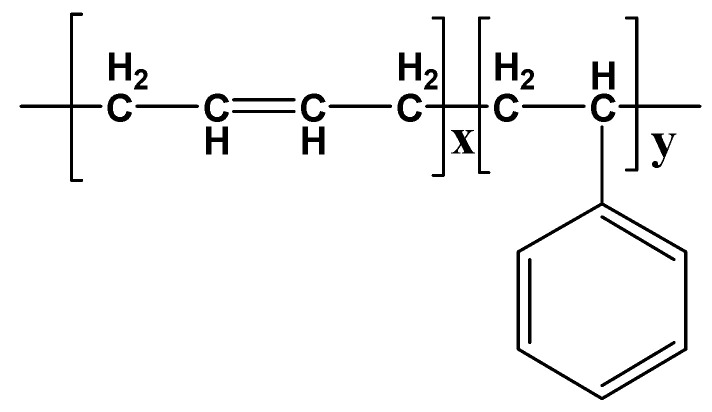
Molecular structure of styrene–butadiene rubber.

Metal-free phthalocyanine (H_2_Pc), carbon nanotubes (CNTs), and styrene–butadiene rubber were used. The phthalocyanine was purchased from Sigma Aldrich, Darmstadt, Germany (Available online: https://www.sigmaaldrich.com/SA/en/product/aldrich/253103, accessed on 29 July 2022), while the multiwalled carbon nanotube (CNT) powder was bought from Sun Nanotek Co., LTD (Nanchang, China). (available online: http://www.sunnano.com/cnt%20product.html, accessed on 12 August 2022). The H_2_Pc powder was used as it was received. The chemical formula of the metal-free phthalocyanine is C_32_H_18_N_8_, while its molecular weight is 514.54. The molecular structure of the H_2_Pc is shown in Figure 1. The multiwalled carbon nanotubes were 100 to 200 nm in length and 10 to 30 nm in diameter. The CNT powder was also used in its as-received form without any processing. Polymeric rubber (styrene–butadiene rubber) was used as a substrate. The molecular structure of the styrene–butadiene rubber is shown in Figure 2.

### 2.2. Methods

**Figure 3:**
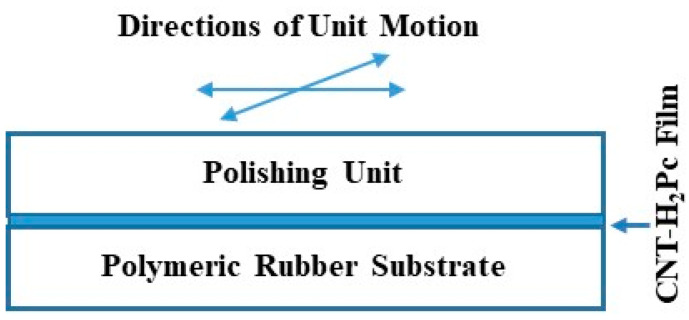
Schematic illustration of the rubbing-in technology used to fabricate the organic H_2_Pc-CNT (metal-free phthalocyanine–carbon nanotubes)-composite-based sensor adapted from Ref. [25].

Two types of deformable semiconductive bolometers, surface-type and sandwich-type sensors, were designed and prepared using the rubber substrate and rubbing-in technology. A schematic diagram illustrating the rubbing-in setup is shown in Figure 3. The dimensions of the polymeric rubber substrates used for the IR bolometer were as follows: 1 × 0.5 × 0.02 cm^3^. Rubbing-in technology, as described above, was used to fabricate the rubber-CNT and rubber-CNT-H_2_Pc composite films. The composite is formed by spreading the powder (H_2_Pc or CNT powder) on the polymeric rubber (styrene–butadiene) substrate in a specified area. The powders are embedded in the surface of the polymeric rubber substrate by rubbing the powders with a solid block. This round-shaped block is made up of metal. This process is carried out using a special mechanism that controls the direction and the frequency of the block. Using this process, the powder penetrates the pores of the rubber and forms a composite layer with the rubber. A rubber-conducting/semiconducting powder composite layer can be formed on both surfaces of the rubber substrate if it is necessary.

Pre-stretching the polymeric rubber substrate prior to rubbing in the conducting or conducting–semiconducting powders enlarges the pores of the rubber and makes the surface more receptive to embedding powered materials, but it makes this procedure more complicated and expensive. In this work, the polymeric rubber substrate’s pre-stretching was circumvented by properly selecting an organic semiconductor material, which is H_2_Pc (metal-free phthalocyanine), and it contains weak Van der Waals forces between the molecules. The procedure of rubbing H_2_Pc into the rubber substrates was realized directly without pre-stretching the substrates, which is one of the technological advantages.

Samples of 1 cm in length, 0.5 cm in width, and 0.02 cm in thickness were prepared using the fabricated setup. To avoid the preliminary stretching of the substrates, the pressure on the powder in the process of fabrication was increased up to 20 gf/cm^2^. The time taken to prepare each sample was 30 s on average. In the middle of the surface-type samples, a mixture of CNT-H_2_Pc powder was deposited as sensitive material, and the ratio of ingredients was 30:70 wt.%. On both sides of the composite layer, the CNT layers were deposited as well. Both CNTs and the CNT-H_2_Pc mixture were incorporated into the rubber substrate using the same procedure (rubbing in). In this process, powders of conducting or semiconducting materials are embedded in polymeric rubber substrates. Because of their flexibility, the polymeric rubber substrates make the devices deformable and shockproof [24].

**Figure 4:**
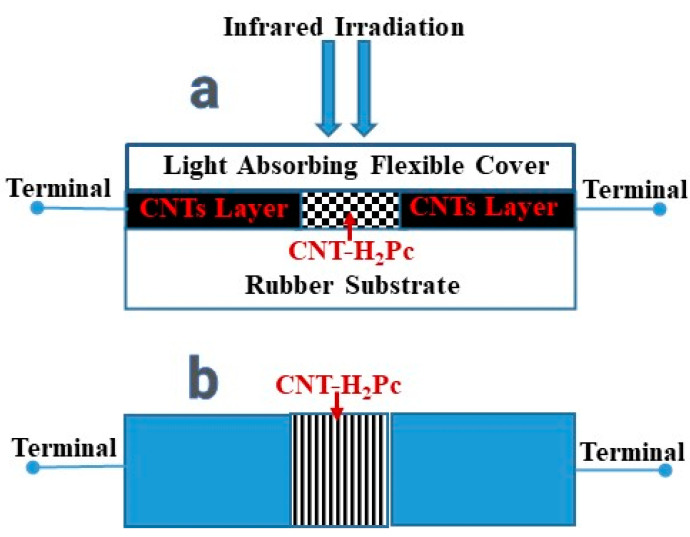
Schematic drawing illustrating the front (**a**) as well as the top (**b**) views of the fabricated shockproof deformable surface-type IR bolometer with an active H_2_Pc-CNT composite layer fabricated by using rubbing-in technology.

The fabricated shockproof and deformable surface-type bolometer is shown in Figure 4. The thicknesses of the CNT and CNT-H_2_Pc layers were 12 ± 2 μm and 19 ± 2 μm, respectively. In the prepared samples, the CNT-H_2_Pc and rubber composite performed the role of sensitive material firstly, and secondly, as a resistive material, the H_2_Pc’s conductivity was significantly lower as compared with the conductivity of the CNTs. The CNT layers were also used as a conductive material because of their high conductivity. The fabricated devices (sensors) showed sufficiently high resistance, which is considered helpful in minimizing and avoiding errors (experimental) that are related to connecting wires and CNT layer resistance.

**Figure 5:**
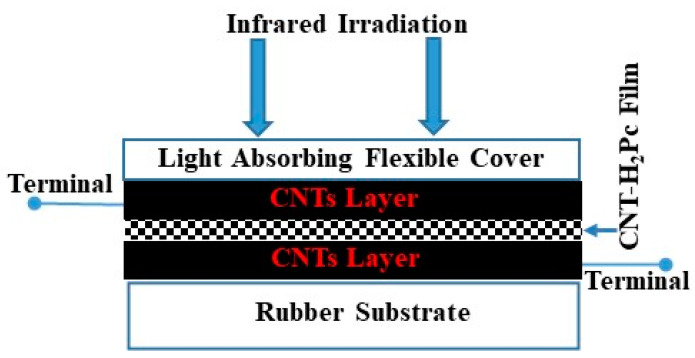
Schematic illustration of the fabricated shockproof deformable sandwich-type IR bolometer with an organic semiconductor layer fabricated by using rubbing-in technology.

Figure 5 shows the schematic drawing (front view) of the fabricated shockproof deformable sandwich-type IR bolometer. The composition of the organic semiconductor layer in the sandwich-type sensors was 30:70 wt.% (CNT:H_2_Pc). The CNT/CNT-H_2_Pc/CNT layer thicknesses were 12 ± 2 μm/19 ± 2 μm/12 ± 2 μm, respectively.

During the characterization of the fabricated bolometers, the impedance (at frequencies of 10 kHz and 200 kHz) and the resistance were measured by using a digital LCR meter (MT 4090). All the experiments were conducted in room temperature conditions. When it was needed, the fabricated sensors were placed in an indigenously designed special chamber. The infrared irradiation was applied to the receiving and largest surface of the sensor. The HP 3616 (PHILIPS), made in Germany, was used as the infrared source. For the measurement of infrared irradiation, an LS122 IR power meter was used. A set of three samples was made for each type of sensor. The results of these sensors were similar to an average variation of ±3.0%. Each sample was tested 3 to 4 times, and the average experimental error was calculated to be up to ±2.5%.

## 3. Results and Discussion

**Figure 6:**
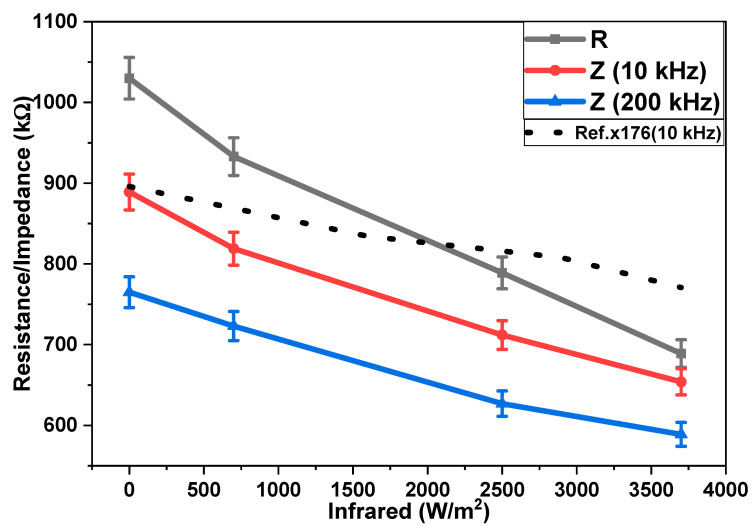
Dependencies of the resistance and the impedances of the surface-type IR bolometer on the intensity of infrared irradiation and their comparison with a reference curve [26]. R = resistance, Z = impedance.

The emission of infrared radiation (in the form of heat) from all matter present on the Earth is well known. Therefore, IR sensing is important not only for the detection of a variety of objects but also for the purposes of monitoring, surveillance, and security. Figure 6 shows the dependencies of the impedances and resistance of the surface-type IR sensor on different infrared irradiation intensities. Impedance was measured at two different frequencies (10 kHz and 200 kHz) under an IR irradiation intensity ranging from 0 to 3700 W/m^2^. Figure 6 shows that, with a rising frequency, a reduction in the impedance of the sensor takes place. It is also shown that the resistance and impedances at 10 kHz and 200 kHz decreased with an increasing intensity of IR irradiation. In the surface-type sensors, decreases in resistance and impedances at 10 kHz and 200 kHz were by 1.49, 1.36, and 1.30 times upon increasing the irradiation intensity from 0 to 3700 W/m^2^. A comparison of impedance–infrared behavior in the surface-type sensor, with previously reported results (reference curve in Figure 6), shows that these sensors have high sensitivity in a similar range of infrared irradiation. These results were compared for the same range of infrared irradiation (0–3700 W/m^2^) at a frequency of 10 kHz.

**Figure 7:**
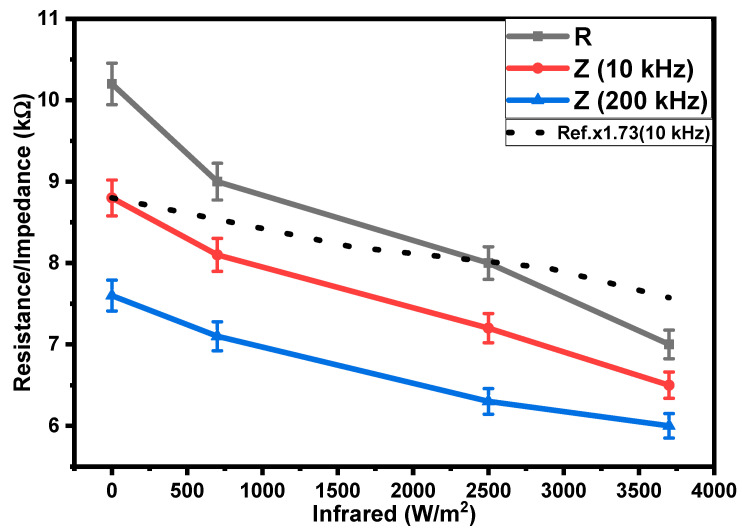
Dependencies of the resistance and the impedance of the sandwich-type IR bolometer on the intensity of infrared irradiation and their comparison with a reference curve [26].

Figure 7 shows that the impedance and resistance of the sandwich-type IR sensor are dependent on the intensity of the infrared irradiation. The impedance was also measured at frequencies of 10 kHz and 200 kHz. The impedance and the resistance of the sandwich-type sensors were also reduced under the influence of IR irradiation (0 to 3700 W/m^2^). By enhancing IR irradiation from 0 to 3700 W/m^2^, the resistance and impedances of the sensors (sandwich-type) at 10 kHz and 200 kHz were reduced by 1.46, 1,35, and 1.27 times. The IR sensitivity of the sandwich-type sensors was better compared with the reported results, as shown in a comparison of solid-line data with a dotted-line reference curve [26]. These results were compared at the same frequency (10 kHz) and under the same range of infrared irradiation (0–3700 W/m^2^).

A comparison of the surface- and sandwich-type bolometers shows that the initial resistance or impedance of the sandwich-type sensors is very low (10.2 kΩ) as compared with the surface-type bolometer (1030 kΩ), while the sensitivity of the surface-type bolometer is very high (92.1 Ωm^2^/W) as compared with the sandwich-type bolometer (0.9 Ω m^2^/W). Physically, this change in impedance and resistance may be due to the heating of the CNT-H_2_Pc composite layer, firstly, and, secondly, due to a rise in the charge concentration and mobility as a result of hopping conduction. Practically, all these phenomena cause a decrease in resistance and increased conductivity, which was noted experimentally and is common in semiconductors [23].

**Figure 8:**
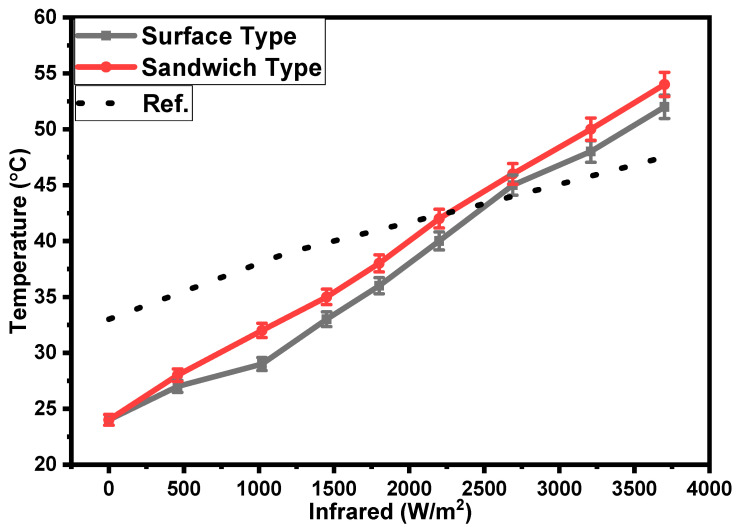
Dependencies of the temperatures of the surface- and sandwich-type sensors on the intensity of infrared irradiation and their comparison with a reference curve [27].

Figure 8 shows the dependencies of the temperatures of the surface- and sandwich-type sensors on the intensity of the IR irradiation. It is shown that, as the intensity of the IR irradiation increases, the surface temperature of the sensors increases as well. The rate of growth in the temperatures of the surface- and sandwich-type samples under the effect of IR irradiation is equal to 0.008 °C/(W/m^2^) on average. This increase in surface temperature is significantly higher as compared with the increase in surface temperature reported in ref. [27]. A comparison of the obtained surface temperature with the reported one is provided in Figure 8. The temperature coefficient of resistance (*TCR*) was calculated for both the sandwich- and surface-type bolometers by using the following equation [20,28,29]:

(1) TCR=dR(R0dT)

where *R*_0_ is the initial resistance, dR is the change in resistance, and dT is the change in temperature. The average values of the *TCR* for the sandwich- and surface-type bolometers are −1.1 and −1.2%/°C, respectively. The values are comparable with the *TCR* values reported in the literature for bolometers. This response can be explained on the basis of the photothermal effect [30], where, upon increasing the temperature of the CNT-H_2_Pc-rubber composite under the influence of infrared irradiation, the electrical resistance/impedance decreases because of negative the *TCR* (temperature coefficient of resistance) of the composite. A comparison of various properties of fabricated bolometers with previously reported bolometers is provided in Table 1.

It is well known that an increase in temperature causes a reduction in the conductivity of metallic materials, but in semiconducting materials, it causes an increase in conductivity (a reduction in resistance) [35]. In this case, the active material is the composite of rubber-H_2_Pc-CNTs, which shows semiconducting properties. Upon increasing the intensity of infrared irradiation, the temperature increases, which generates charge carriers. Because of the increase in the concentration of charge carriers, the resistance decreases. The mobility also increases because of a decrease in the potential barrier and the hopping length caused by increasing carrier density. Likewise, polaronic conduction may also explain conduction between neighboring sites that are separated by energy barriers [36,37].

One aspect of this study is that it provides a deformable, semiconductive composite made using rubbing-in technology. This technology permits the formation of regions in a rubber substrate. Some regions may be doped with organic semiconducting material (such as H_2_Pc), while the other regions may be doped with conductive material (such as CNTs). The doping may be conducted in the form of layers or patterns. The rubber (styrene–butadiene rubber) substrates, which have the ability to deform or compress, were used for sensor fabrication. The organic or inorganic conductive or semiconductive materials may be embedded on one or both surfaces of the substrate using rubbing-in technology. The same substrate may be patterned on opposite sides with the same or different electrical patterns to enhance the efficiency of the device. Such types of devices may be used in a variety of applications without limitation. Moreover, the compression and deformation of such devices negligibly affect their electrical performance [38].

The experimental results shown in Figure 6, Figure 7 and Figure 8 may be simulated by using the following mathematical function [39]:

(2) $$f\left(x\right)=e^{x}$$

This is linearized with the help of a natural logarithm to represent the curvilinear nature of the experimental relationship. A log-linear form of Equation (2) is used for the simulation.

**Figure 9:**
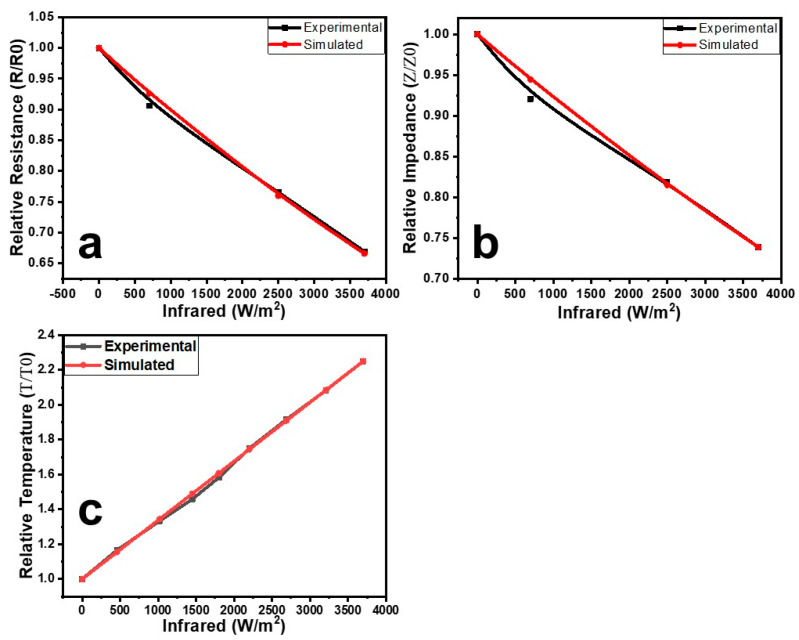
Comparison of the experimental and simulated resistance–infrared irradiation relationships of the surface-type sensors (**a**), the impedance–infrared irradiation relationships of the sandwich-type sensors (**b**), and the temperature–infrared irradiation relationships of the sandwich-type sensors (**c**).

For the results of surface- and sandwich-type sensors, some results were simulated by using a log-linear form of the above function (Equation (2)). The relative resistance–infrared irradiation relationship of the surface-type sensors was simulated by using a modified form of the above function. For the compression, the above function (Equation (2)) may be represented as follows (Equation (3)):

(3) RR0=eIirk1

where *R* and *R*_0_ are the instantaneous and initial resistances of the surface-type sensor. *I_ir_* is the instantaneous intensity of infrared irradiation. *k*_1_ is the resistance–infrared irradiation coefficient, and its value was calculated as −1.09 × 10^−4^ m^2^/W with 95% confidence boundaries of −1.18 × 10^−4^ and −9.93 × 10^−5^ and a *p*-value of 0.0000, which shows the statistical significance and reliability of the estimates. The R^2^ value of this estimate is 0.994253, which indicates the high explanatory power of IR in determining *R/R*_0_. Figure 9a shows a comparison of the experimental and simulated results of the resistance–infrared irradiation relationships of the surface-type sensors. It can be seen in Figure 9a that the experimental and simulated results are well matched. The impedance–infrared irradiation relationships shown in Figure 6 can also be simulated by using the same approach.

The impedance–infrared irradiation relationship of the sandwich-type sensor (shown in Figure 7) was simulated by using a modified version of the mathematical function (Equation (2)). The modified mathematical function is shown in Equation (4).

(4) ZZ0=eIirk2

where *Z* and *Z*_0_ are the instantaneous and initial impedances of the sandwich-type sensor. The *k*_2_ is the impedance–infrared irradiation coefficient, and its value is −8.19 × 10^−5^ m^2^/W with 95% confidence boundaries of −9.28 × 10^−5^ and −7.18 × 10^−5^ and a *p*-value of 0.0000, which shows the statistical significance and reliability of the estimates. The R^2^ value of this estimate is 0.987388, which indicates the high explanatory power of IR in determining *Z/Z*_0_. A comparison of the simulated and experimental results is shown in Figure 9b. The simulated and experimental results are well matched with each other.

The results shown In Figure 8 can be simulated by using the following linear function [39]:

(5) $$f\left(x\right)=ax+b$$

To simulate the temperature–infrared irradiation relationship, the above function (Equation (5)) may be modified as follows:

(6) TT0=Iirk3

where *T* and *T*_0_ are the instantaneous and initial temperatures of the sandwich-type sensor. *k*_3_ is the temperature–infrared irradiation factor, and its value is +3.38 × 10^−4^ m^2^/W with 95% confidence boundaries of +3.28 × 10^−4^ and +3.5 × 10^−4^ and a *p*-value of 0.0000, which shows the statistical significance and reliability of the estimates. The R^2^ value of this estimate is 0.998746, which indicates the high explanatory power of IR in determining *T/T*_0_. *I_irm_* is the maximum intensity of infrared irradiation. A comparison of the experimental and simulated results is shown in Figure 9c. The simulated results (Figure 9c) are in good agreement with the experimental results.

## 4. Conclusions

In this work, shockproof deformable H_2_Pc (organic semiconductor)–CNT-composite-based sensors were fabricated on polymeric rubber substrates. The fabricated sensors were investigated for IR irradiation sensing. The sensing mechanism was based on the change in impedance and resistance with the variation in IR intensity. The novel ratio of the H_2_Pc-CNT composite ingredients and comparably high value of the *TCR* make the devices attractive for bolometric applications meant to measure the intensity of infrared radiation. Because of their easy fabrication, low cost, and high sensitivity, the fabricated sensors are very attractive for commercialization.

## Figures and Tables

**Table 1:** Comparison of various properties of fabricated bolometers with previously reported bolometers.

Sr. #	Sensing Materials	Fabrication Technology	Device Design	*TCR* (%/K)	Sensitivity	Ref.
1	Single-crystal Si	SOI Process	Diode	0.5–0.7	-	[31]
2	SWCNT composite	High-pressure method	Bolometer	−2.94	1.2 × 10^8^ cm Hz^1/2^/W	[20]
3	Polycrystalline silicon	CMOS process	Microbolometer	−0.52	218.2 Ω/°C	[32]
4	MoS_2_-CNT composite	Facile hydrothermal process	-	0.25	-	[33]
5	Polysilicon	CMOS process	Infrared microbolometer	0.34 to 0.58	2.2 × 10^8^ cm Hz^1/2^/W	[34]
6	CNT-H_2_Pc	Rubbing-in technology	Sandwich-type bolometer	−1.1	0.87 Ω/(W/m^2^)	Current Study
			Surface-type bolometer	−1.2	−92.2 Ω/(W/m^2^)	

SOI: silicon on insulator, CMOS: complementary metal–oxide–semiconductor, SWCNT: single-walled carbon nanotube.

## Data Availability

The data presented in this study are available on request from the corresponding author.
